# Salvage Autologous Stem Cell Transplantation in Daratumumab-Refractory Multiple Myeloma

**DOI:** 10.3390/cancers13164019

**Published:** 2021-08-10

**Authors:** Lakshmi Yarlagadda, Sravani Gundarlapalli, Richa Parikh, Reid D. Landes, Mathew Kottarathara, Yetunde Ogunsesan, Shadiqul Hoque, Angel A. Mitma, Clyde Bailey, Kerri M. Hill, Sharmilan Thanendrarajan, Monica Graziutti, Meera Mohan, Maurizio Zangari, Frits van Rhee, Guido Tricot, Carolina Schinke

**Affiliations:** 1 Myeloma Center, Division of Hematology/Oncology, Winthrop P. Rockefeller Cancer Institute, University of Arkansas for Medical Sciences, Little Rock, AR 72205, USA; lsyarlagadda@uams.edu (L.Y.); sgunarlapalli@uams.edu (S.G.); rparikh@uams.edu (R.P.); mkottarathara@uams.edu (M.K.); YOgunsesan@uams.edu (Y.O.); mshoque@uams.edu (S.H.); amitma@uams.edu (A.A.M.); baileyclyde@uams.edu (C.B.); HillKerriM@uams.edu (K.M.H.); sthanendrarajan@uams.edu (S.T.); mgraziutti@uams.edu (M.G.); mzangari@uams.edu (M.Z.); vanrheefrits@uams.edu (F.v.R.); gtricot@uams.edu (G.T.); 2 Department of Biostatistics, University of Arkansas for Medical Sciences, Little Rock, AR 72205, USA; rdlandes@uams.edu; 3 Cancer Center, Division of Hematology/Oncology, Medical College of Wisconsin, Milwaukee, WI 53226, USA; memohan@mcw.edu

**Keywords:** multiple myeloma, daratumumab, autologous stem cell transplantation

## Abstract

**Simple Summary:**

Multiple Myeloma (MM) is the most common cancer of the bone marrow and remains incurable despite advances in novel therapy. The disease course is typically characterized by an initial response pattern to treatment followed by eventual relapse and treatment refractoriness. Patients who have progressed on several novel therapies, including the CD38-targeting monoclonal antibody Daratumumab, have a dismal outcome with a median overall survival of less than 10 months and are in dire need of therapies with new mechanisms. While emerging novel modalities have shown promising results, the current study explores the use of high-dose chemotherapy followed by autologous stem cell transplantation (ASCT) in heavily pretreated Daratumumab-refractory MM patients. Our results for 69 consecutive patients treated with salvage ASCT indicate that this approach can lead to long-term MM control and should be considered a treatment modality in selected heavily pretreated Daratumumab-refractory patients.

**Abstract:**

Daratumumab, a CD38-targeting monoclonal antibody, has significantly improved survival rates in multiple myeloma (MM), yet patients who progress on Daratumumab have dismal clinical outcomes with an overall median of less than 10 months. While emerging novel modalities have shown promising results, the current study explores the use of high-dose chemotherapy followed by autologous stem cell transplantation (ASCT) in heavily pretreated Daratumumab-refractory MM patients. We retrospectively investigated the outcome of 69 consecutive patients who received upfront ASCT. The median progression-free survival (PFS) for the entire patient cohort was 7.2 months with a median overall survival (OS) of 19.3 months. For patients with ≥very good partial response (VGPR), median PFS and OS improved to 9 months and 34 months, respectively. Achievement of MRD negativity in ≥VGPR did not further improve the outcome. A better performance status, younger age, longer time interval from initial MM diagnosis/initial ASCT to salvage ASCT and low-risk GEP70 were all associated with improved PFS and OS after salvage ASCT. Our results suggest a role for salvage ASCT in selected heavily pretreated and Daratumumab-refractory patients.

## 1. Introduction

Novel therapies, such as immunomodulatory drugs (IMiDs), proteasome inhibitors (PIs), and monoclonal Antibodies (moAbs) have significantly improved the depth of response and clinical outcome in MM [1,2]. However, most patients will eventually relapse and develop refractory disease. Recent multicenter studies have focused on the outcome of patient groups with refractoriness to certain novel drug classes as contemporary benchmarks to identify those with the highest need for therapies based on entirely novel mechanisms [3,4]. Patients with double refractoriness to PIs and IMiDs portend poor outcomes with a median overall survival (OS) of approx. 13 months [5]. Monoclonal antibodies (moAbs) targeting CD38, such as Daratumumab and Isatuximab, have profoundly improved outcome in relapsed refractory MM (RRMM) and have shown impressive activity as a single agent and in combination with IMiDs and PIs [6,7,8,9,10]. Not surprisingly, patients who progress on CD38 moAbs have dismal outcomes, with a recent meta-analysis showing the median OS to be only 8.6 months in this patient population [3]. Patients who are “penta-refractory” (refractory to 2 Pis, 2 IMiDs, and CD38 moAbs) tend to fare even worse with a median OS of only 5.6 months [3]. While new classes of drugs, particularly immunotherapy with CAR-T cells and bispecific antibodies, have shown promising results, their efficacy in this particular refractory patient group remains to be established. Even for those who received upfront ASCT, salvage autologous stem cell transplantation (ASCT) for patients with relapsed MM has shown to be effective and feasible, with median PFS ranging from 6 to 18 months and median OS ranging from 15 to 56 months [11,12,13,14,15,16,17,18,19,20]. The different outcomes for these trials are likely due to differences in patient selection and delays in salvage ASCT in favor of novel therapies. The efficacy of salvage ASCT in patients who have become refractory to moAbs has, to our knowledge, not been assessed yet and is of high clinical relevance to offer potential therapeutic alternatives in this highly pretreated population. Hence, to determine the impact of salvage ASCT on clinical outcome in CD38 moAb refractory patients, we investigated the outcomes of 69 patients who had progressed on Daratumumab and subsequently underwent a salvage ASCT.

## 2. Materials and Methods

### 2.1. Patients

We conducted a retrospective chart review on 69 consecutive patients who received salvage ASCT after MM progression on Daratumumab and extensive prior exposure to other novel agents. As previously reported by Ghandi et al., Daratumumab-refractoriness was defined as having received at least four weeks of Daratumumab and showing signs of progression by IMWG criteria [3,21]. Furthermore, all patients received upfront Melphalan-based stem cell transplantation at diagnosis with either single or tandem ASCT and subsequently progressed. Median time from initial ASCT (or tandem ASCT) to salvage ASCT was short at 36.4 months (5–169 months). 

The preparative regimens for salvage ASCT were either: (1) Melphalan-based therapy with patients receiving either 200 mg/m^2^ or 140 mg/m^2^ of Melphalan, or (2) BEAM-based therapy (Carmustine, Etoposide, Cytarabine, and Melphalan) or (3) VDT-PACE (Velcade, Dexamethasone, Thalidomide, Cisplatin, Adriamycin, Cyclophosphamide, and Etoposide) with low-dose Melphalan (80 mg/m^2^). The choice of the myeloablative regimen was decided by the treating oncologist upon careful consideration of age, performance status, and organ function. The study was approved by the University of Arkansas for Medical Sciences IRB and performed in accordance with the Declaration of Helsinki.

### 2.2. Response Assessment and Risk Stratification

The response to salvage ASCT was evaluated within 100 days using IMWG criteria [22]. Imaging analysis using PET-CT or DW-MRI was performed as previously described to document the response of focal lesions [23]. MRD was assessed using eight-color flow cytometry as previously described [24]. In brief, BM samples were immunephenotyped on a FACSCanto II flow cytometer using an eight-color technique (CD138 (V-500), CD38 (FITC), CD19 (PE-Cy7), CD45 (V-450), CD27 (PercpCy5.5), CD81 (APC-H-7), CD56 (APC), and CD20 (PE)). MRD negativity was defined by the presence of fewer than 20 events indicating phenotypically aberrant clonal plasma cells after acquiring at least 2.0 × 10^6^ total events. MRD sensitivity was one MM cell in 10^5^ bone marrow cells. GEP70 risk stratification was analyzed on available samples at diagnosis as previously published using Affymetrix U133 2.0 plus arrays (Affymetrix, Santa Clara, CA, USA) on CD138-enriched PCs [25,26]. 

### 2.3. Statistical Analysis

We estimated median survival times with Kaplan–Meier’s method (product-limit survival) and, when comparing survival between two independent groups, using log-rank $\chi^{2}$ tests. When evaluating whether select clinical factors (mostly continuous) were associated with risks of progression and death, we used Cox proportional hazards regression to estimate hazard ratios. We note that a Cox proportional hazards regression is asymptotically equivalent to the log-rank $\chi^{2}$ test when the only factor is categorical. The significance level was 0.05; however, where possible, we present 95% confidence intervals rather than *p*-values.

## 3. Results

### 3.1. Patient Characteristics

The median age of the patient cohort at a time point of salvage ASCT was 69 years (39–79), with 65% of the cohort being male and 81% Caucasian, Table 1. GEP70 risk signature, assessed at diagnosis, classified 32% of patients as high-risk; 23% had no risk score available. All patients received at least one upfront ASCT, and 58% had tandem ASCT (defined as two consecutive ASCTs within 6 months). Median lines of therapies prior to salvage ASCT were five (range: 3–14), with all patients having progressed while on Daratumumab and the vast majority having been exposed to all three IMiDs (Thalidomide: 99%, Lenalidomide: 95%, and Pomalidomide: 86%) and at least two PIs (Bortezomib: 99%, Carfilzomib: 88%, and Ixazomib: 23%). A BEAM-based conditioning regimen was used most frequently for salvage ASCT (43%), followed by high dose Melphalan (28%) and low-dose Melphalan in combination with PACE chemotherapy (28%). The median number of CD34 cells infused was 7.5 × 10^6^/kg (1.6–15 × 10^6^/kg). After response assessment within 100 days of salvage ASCT, patients began maintenance treatment mostly with combinations of previously used novel agents, including Thalidomide (16%), Lenalidomide (6%), Pomalidomide (32%), Bortezomib (13%), and Carfilzomib (20%). Additionally, 26% of patients were re-exposed to Daratumumab despite having developed refractoriness prior to salvage ASCT. Cyclophosphamide was also commonly provided as part of the maintenance therapy after salvage ASCT (26%). Newer drugs such as Selinexor were only administered recently after FDA approval. The most common combinations used were Daratumumab/Pomalidomide/Dexamethasone (17.4%), Pomalidomide/Cyclophosphamide/Dexamethasone (14.5%), Carfilzomib with Dexamethasone (9%), and Carfilzomib/Pomalidomide/Dexamethasone (6%).

### 3.2. Efficacy

**Figure 1:**
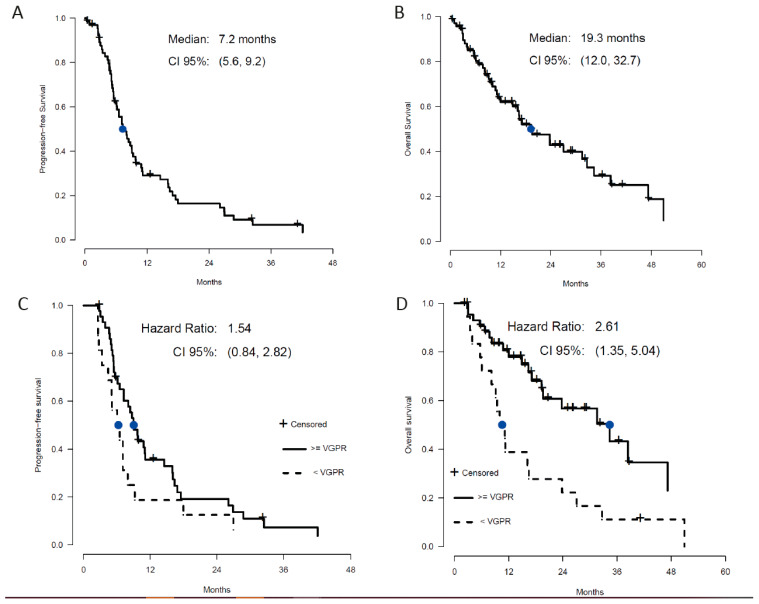
Median PFS (**A**) and OS (**B**) for the entire patient cohort. Clinical outcome stratified by response shows significantly better PFS (**C**) and OS (**D**) in patients who at least achieved a VGPR.

The 100-day mortality post-salvage transplant was 10% (7/69), with four out of seven patients already showing disease progression, suggesting that mortality was not solely treatment-related. Post-ASCT response was assessed in 91% (63/69) of patients within 100 days of ASCT. The overall response rate was 80% (45/69) with a CR in 43% (30/69), a VGPR in 21% (15/69), and a PR in 15% (10/69), Table 1. Stable disease was seen in 3% (2/69) and progressive disease in 9% (6/69) of patients. MRD negativity was attained in 45% (31/69) of patients; all but two patients (PR) had at least a clinical VGPR. With a median follow-up of 14 months, median PFS for the whole cohort was 7.3 months, with 13% not progressing at the last follow-up (Figure 1A), and median OS was 19.3 months with 42% alive at the last follow up (Figure 1B). Comparing those who had at least a VGPR versus those with less than a VGPR (total *n* = 63), median PFS was not statistically different (9.0 vs. 6.8 months), but the OS was significantly better in patients with at least a VGPR (34.4 vs. 10.6 months; *p* = 0.004), Figure 1C,D. Considering only those with VGPR or better (total *n* = 40), MRD negativity did not further improve PFS or OS as might be expected, but the curves essentially overlapped with no significant difference (Figure S1). Across all 69 patients, 25% of patients were projected to be alive > 36 months after salvage ASCT, with 12% (8/69) known to have survived that long.

### 3.3. Clinical Parameters Predictive of Response after Salvage ASCT

**Figure 2:**
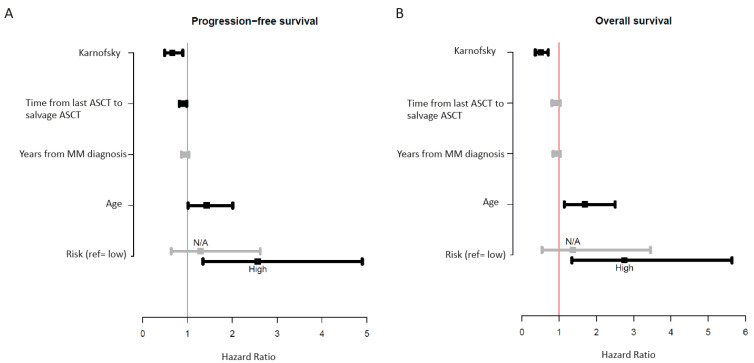
Univariate analysis of clinical markers associated with improved or worse PFS (**A**) and OS (**B**).

We then individually examined clinical parameters for an association with improved survival after salvage ASCT (Figure 2A,B). Clinical parameters significantly associated with inferior outcome were increasing age (PFS *p* ≤ 0.05, OS *p* ≤ 0.01), poor performance status (PFS *p* ≤ 0.01, OS *p* ≤ 0.0001), and high GEP70 risk score at diagnosis (PFS *p* ≤ 0.01, OS *p* ≤ 0.01). An increased time interval from initial ASCT (measured from either single ASCT or the second ASCT if performed in a tandem fashion) to salvage ASCT showed an improved outcome but was only significant for PFS (*p* ≤ 0.05). There was a nonsignificant trend for inferior outcomes with increased preceding therapy lines. The choice of the conditioning regimen (BEAM vs. Melphalan vs. low-dose Melphalan with hybrid chemotherapy) did not significantly impact PFS or OS in this patient cohort.

## 4. Discussion

To our knowledge, our study is the first to address the role of salvage ASCT in patients with universal exposures to multiple novel agents and refractoriness to Daratumumab. We show that high-dose chemotherapy followed by ASCT achieves substantial responses in 80% of patients, with an estimated 25% of these patients projected to be alive at 36 months. Although the present study is limited by a relatively small patient size, it highlights a role for salvage ASCT in selected heavily pretreated patients and compares favorably with previously published trials [3,4]. The response rates and survival outcome appear to be comparable to recent data with novel Car T cell treatment [27], suggesting that salvage ASCT constitutes a valid alternative in selected patients. While maintenance post-salvage ASCT may have influenced the PFS and OS in this cohort, the impact of the regimens used in maintenance has shown to be rather small in this refractory patient population with short PFS and OS [3,4], emphasizing that salvage ASCT contributes majorly to the improved outcome. It is of interest that while patients who achieved at least a VGPR showed a longer PFS and OS, achievement of MRD negativity at a level of one MM cell in 10^5^ had no further impact on the outcome. The reasons for that are not entirely clear but can be explained, at least in part, by the commonly observed macrofocal nature of relapse in patients with late-stage disease. This pattern is associated with active disease in focal lesions or at extramedullary sites, while there is no MM infiltration of random bone marrow, and hence MRD negativity in these patients is not a useful prognostic tool [23]. Furthermore, previous studies have shown shorter PFS after initial transplant as a strong, adverse prognostic marker for PFS and OS after salvage ASCT [14,17,28]. Although we did not have data on PFS after initial ASCT available in this cohort, we show that a shorter time frame from initial ASCT to salvage ASCT predicts for worse outcome after salvage ASCT; however, this was only significant for PFS, which may be due to the limited follow-up. Other clinical markers that were significantly associated with adverse outcomes after salvage ASCT included worse performance status, older age, and a high GEP70 risk score at diagnosis. The observation that the GEP70 score could distinguish patients with significantly worse PFS and OS in this overall heavily pretreated and refractory population confirms its overall prognostic significance and suggests that this patient category might be more heterogeneous than previously anticipated. It also must be highlighted that salvage ASCT not only leads to drastic cytoreduction in the majority of patients but can also restore robust hematopoiesis as previously reported by Tremblay et al. [11]. The ability to correct cytopenias in this heavily pretreated patient population is of critical value as low blood counts often preclude patients from continuing novel therapies or being enrolled in clinical trials. It is, therefore, important to underscore that salvage ASCT in selected patients can also be used as a means of bridging before proceeding to novel therapies.

## 5. Conclusions 

Our results suggest a role for salvage ASCT in selected heavily pretreated patients, albeit there remains an apparent clinical need for novel therapies. With the rapidly increasing proportion of heavily pretreated, Daratumumab-refractory patients and the emergence of novel effective therapies, further clinical studies will be necessary to clarify which treatment modality will yield the best outcome for individual patients.

## Figures and Tables

**Table 1:** Patient characteristics.

Characteristic	Value (*n* = 69)
Median Age at salvage ASCT (range)—years	61 (39–79)
Male—% (No.)	65% (45/69)
Race—% (No.)	
Caucasian	81% (56/69)
African American	17% (12/69)
Native American	1% (1/69)
GEP 70 risk score at diagnosis—% (No.)	
High-risk	32% (22/69)
Low-risk	45% (31/69)
Unknown	23% (16/69)
Karnosfky ≥ 90—% (No.)	58% (40/69)
Median lines of prior therapy (min-max)	5 (3–14)
Upfront ASCT—% (No.)	100% (69/69)
Upfront tandem ASCT *—% (No.)	58% (40/69)
Prior exposure to—% (No.)	
Daratumumab (refractory)	100% (69/69)
Velcade	99% (68/69)
Carfilzomib	88% (61/69)
Ninlaro	23% (16/69)
Thalidomide	99% (68/69)
Lenalidomide	95% (66/69)
Pomalyst	86% (59/69)
Median hemoglobin at relapse, g/dL (min–max)	9.5 (7.5–12.3)
Median creatinine at relapse, mg/dL (min–max)	1 (0.4–3.4)
Conditioning regimen for salvage ASCT—% (No.)	
BEAM-based	43% (30/69)
Melphalan-based	28% (20/69)
Low-dose Melphalan with hybrid chemotherapy	28% (19/69)
Best response after salvage ASCT—% (No.)	
sCR/CR	43% (30/69)
VGPR	21% (15/69)
PR	15% (10/69)
SD	3% (2/69)
PD	9% (6/69)
NA	7% (5/69)
MRD negative after salvage ASCT ^§^—% (No.)	45% (31/69)
Maintenance regimen included—% (No.)	
Pomalidomide	32% (22/69)
Thalidomide	16% (11/69)
Lenalidomide	6% (4/69)
Carfilzomib	20% (14/69)
Bortezomib	13% (9/69)
Daratumumab	26% (18/69)
Cyclophosphamide	26% (18/69)
Selinexor	3% (2/69)

* within 6 months of first ASCT. ^§^ MRD measured with 8 color flow cytometry to a sensitivity of 10^−5^.

## Data Availability

The data presented in this study are available on request from the corresponding author.

## Supplementary Materials

The following are available online at https://www.mdpi.com/article/10.3390/cancers13164019/s1, Figure S1: shows PFS (A) and OS (B) for patients with at least a VGPR who are MRD positive or negative at one cell in 10^5^.
