# Supplementary material for: Salvage Autologous Stem Cell Transplantation in Daratumumab-Refractory Multiple Myeloma
Source: Cancers (Basel). 2021 Aug 10;13(16):4019. doi: 10.3390/cancers13164019 (PMC8392190; doi:10.3390/cancers13164019)
Supplement: Supplementary file 1 [file cancers-13-04019-s001.zip › cancers-1309784-supplementary.pdf]

Supplemental Figure S1

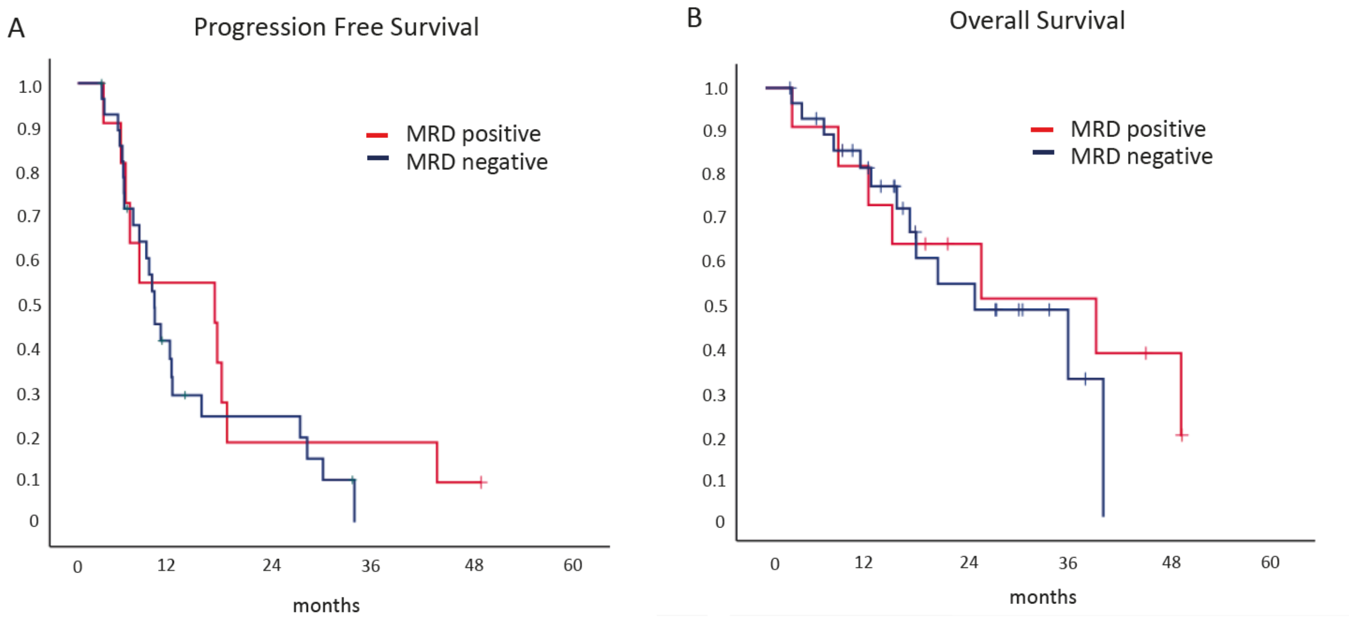

Supplemental Figure S1. shows PFS (A) and OS (B) for patients with at least a VGPR who are MRD positive or negative at one cell in 10^5. There was no significant difference.
